# Acute experimental infection of bats and ferrets with Hendra virus: Insights into the early host response of the reservoir host and susceptible model species

**DOI:** 10.1371/journal.ppat.1008412

**Published:** 2020-03-30

**Authors:** Amanda P. Woon, Victoria Boyd, Shawn Todd, Ina Smith, Reuben Klein, Isaac B. Woodhouse, Sarah Riddell, Gary Crameri, John Bingham, Lin-Fa Wang, Anthony W. Purcell, Deborah Middleton, Michelle L. Baker

**Affiliations:** 1 Department of Biochemistry and Molecular Biology and Infection and Immunity Program, Biomedicine Discovery Institute, Monash University, Clayton, Victoria, Australia; 2 Immunocore Ltd, Abingdon, Oxford, United Kingdom; 3 CSIRO Health and Biosecurity Business Unit, Australian Animal Health Laboratory, Geelong, Victoria, Australia; 4 Medical Research Council (MRC) Human Immunology Unit, MRC Weatherall Institute of Molecular Medicine (WIMM), John Radcliffe Hospital, University of Oxford, Oxford, United Kingdom; 5 Centre of Innate Immunity and Infectious Diseases, Hudson Institute of Medical Search, Clayton, Victoria, Australia; 6 CSIRO, Australian Animal Health Laboratory, Geelong, Victoria, Australia; 7 Programme in Emerging Infectious Diseases, Duke–National University of Singapore Medical School, Singapore; Division of Clinical Research, UNITED STATES

## Abstract

Bats are the natural reservoir host for a number of zoonotic viruses, including Hendra virus (HeV) which causes severe clinical disease in humans and other susceptible hosts. Our understanding of the ability of bats to avoid clinical disease following infection with viruses such as HeV has come predominantly from *in vitro* studies focusing on innate immunity. Information on the early host response to infection *in vivo* is lacking and there is no comparative data on responses in bats compared with animals that succumb to disease. In this study, we examined the sites of HeV replication and the immune response of infected Australian black flying foxes and ferrets at 12, 36 and 60 hours post exposure (hpe). Viral antigen was detected at 60 hpe in bats and was confined to the lungs whereas in ferrets there was evidence of widespread viral RNA and antigen by 60 hpe. The mRNA expression of *IFN*s revealed antagonism of type I and III *IFN*s and a significant increase in the chemokine, *CXCL10*, in bat lung and spleen following infection. In ferrets, there was an increase in the transcription of *IFN* in the spleen following infection. Liquid chromatography tandem mass spectrometry (LC-MS/MS) on lung tissue from bats and ferrets was performed at 0 and 60 hpe to obtain a global overview of viral and host protein expression. Gene Ontology (GO) enrichment analysis of immune pathways revealed that six pathways, including a number involved in cell mediated immunity were more likely to be upregulated in bat lung compared to ferrets. GO analysis also revealed enrichment of the type I IFN signaling pathway in bats and ferrets. This study contributes important comparative data on differences in the dissemination of HeV and the first to provide comparative data on the activation of immune pathways in bats and ferrets *in vivo* following infection.

## Author summary

Bats are natural reservoirs for a number of viruses, including HeV that cause severe disease in humans and other susceptible hosts. We examined acute HeV infection in pteropid bats, compared to ferrets, a species that develops fulminating disease following exposure to HeV, similar to humans. Analysis of HeV replication and transcription of innate immune genes was performed at 12, 36 and 60 hpe and global proteomics was performed on tissues at 60 hpe to obtain insight into the mechanisms responsible for innocuous (bats) compared to fatal (ferrets) HeV infection. We confirmed that both animal species had become infected on the basis of detection of viral RNA in bat lung (60 hpe) and ferret lung, lymph node, spleen, heart and intestine (36 and/or 60 hpe). Analysis of the transcription of *IFN*s and *CXCL10*, combined with global proteomics analysis revealed differences in the activation of the immune response between bats and ferrets, consistent with the difference in the control of viral replication and the development of pathology associated with disease between the two species. This study represents the first *in vivo* comparison between bats and a susceptible host and contributes important information on the kinetics and control of HeV in these two model species.

## Introduction

Bats have attracted increasing attention since being recognized as the source of numerous emerging and re-emerging viruses, including some that are highly pathogenic to other mammals. Remarkably, with the exception of the rhabdoviruses; rabies and lyssaviruses and the arenavirus; Tacaribe virus, bats develop no clinical signs of disease despite the presence of virus in tissues [1,2,3]. Among the viruses that bats coexist with in the absence of disease are the henipaviruses; Hendra virus (HeV) and Nipah virus (NiV) which are carried by flying foxes (*Pteropus* spp.) [4,5,6,7]. Spillover of HeV from flying foxes to horses and then into humans has occurred regularly since 1994, with 94 horse cases and seven human cases reported to 2015 and spillover events continuing to be recorded annually [8,9]. A similar situation exists for the Malaysian strain of the closely related NiV, which has been reported to spillover from bats into pigs and subsequently into humans. In contrast, the Bangladesh strain of NiV shows indirect bat-to-human transmission through contact with food or surfaces contaminated with infectious material from bat saliva, urine or feces and direct human-to-human transmission [10]. Henipavirus infections in humans are characterized by an influenza-like illness that may progress to pneumonia and encephalitis with mortality rates of between 50–100% [11].

Experimental henipavirus infections of pteropid bats are subclinical with transient detection of virus or viral genome in tissues and inconsistent development of neutralizing antibody. Inflammatory and degenerative changes are limited, and have been confined to occasional blood vessels. Sporadic positive immunostaining for HeV has been recorded in blood vessels of spleen, kidney, meninges and placenta of some bats following parenteral exposure, and virus has been re-isolated from tissues including heart, buffy coat, kidney and spleen [12,13].

In contrast to bats, the domestic ferret (*Mustela putorius furo*) reliably develops fulminating disease following exposure to either HeV or NiV, with principal involvement of the vascular system and involvement of multiple organ systems including the lung, lymphoid tissues and central nervous system [14,15,16]. Although the lack of ferret specific phenotyping reagents has limited investigations into the immune response of ferrets, the use of ferrets as a model for influenza virus infection has resulted in the development of a variety of real time PCR assays to quantify mRNA levels of immune genes [17]. Proteomics has also been used to evaluate changes in protein expression during disease pathogenesis in ferrets [18,19,20].

Our previous studies have demonstrated that tissues and cells from uninfected pteropid bats constitutively express the interferon alpha gene (*IFNA*), providing a heightened level of immune activation compared with other species [21]. In view of the key role of IFNs in the innate immune response, we hypothesized that the ability of bats to control viral infection may be associated with the ability of the innate immune response to control viral replication more rapidly compared to species that succumb to disease. The aim of this study was to obtain information on the early response to viral infection in the pteropid bat species, the Australian black flying fox as compared to ferrets, in order to characterize properties of the innate immune response that may be associated with innocuous (bats) versus fatal (ferrets) infection outcomes. Because few bat- and ferret- specific reagents exist, we performed whole proteome analysis of lung tissues from experimentally infected bats and ferrets to obtain a global profile of host and virus protein expression. To our knowledge, this is the first comparative proteomics analysis of experimentally infected bat and ferret tissues. Although some proteomics data has been generated for ferrets, our datasets represent some of the largest proteomics datasets for both species [20]. Our results demonstrate differences in the pattern of viral replication between pteropid bats and ferrets. The global protein expression profile varied between individual bats and ferrets, with GO immune pathway analysis revealing the enrichment of proteins involved in type I IFN signaling in bats and ferrets and evidence for an increased signature of upregulation of cell mediated immune pathways in bats. Transcription of type I and III *IFNs* and C-X-C motif chemokine 10 (*CXCL10)* genes also revealed species specific expression patterns that may be associated with the differences in the outcome of infection.

## Results

Animal identification numbers used throughout the text reflect the species and euthanasia timepoint. For bats: B1-0, B2-0 (0 hpe), B3-12, B4-12, B5-12 (12 hpe), B6-36, B7-36 (36 hpe) and B8-60, B9-60 (60 hpe). For ferrets: F1-0, F2-0 (0 hpe), F3-12, F4-12, F5-12 (12 hpe), F6-36, F7-36 (36 hpe) and F8-60, F9-60 (60 hpe).

### Clinical observations

Bats: All bats remained clinically healthy throughout the study period. Bodyweights at euthanasia were within 10% of pre-challenge weights, reflecting either a mild increase or decrease over the study period. No febrile responses were recorded in any of the bats following HeV infection.

Ferrets: As expected from the short duration of the study, all ferrets remained clinically healthy throughout the study period. Bodyweights at euthanasia were within 10% of pre-challenge weights, reflecting either a mild increase or decrease over the study period. No febrile responses were recorded for any of the ferrets following HeV infection.

### Detection of HeV RNA in swabs and urine

Bats: Viral RNA was not detected in oral, nasal or rectal swabs, nor in the urine samples from any bat exposed to HeV.

**Fig 1:**
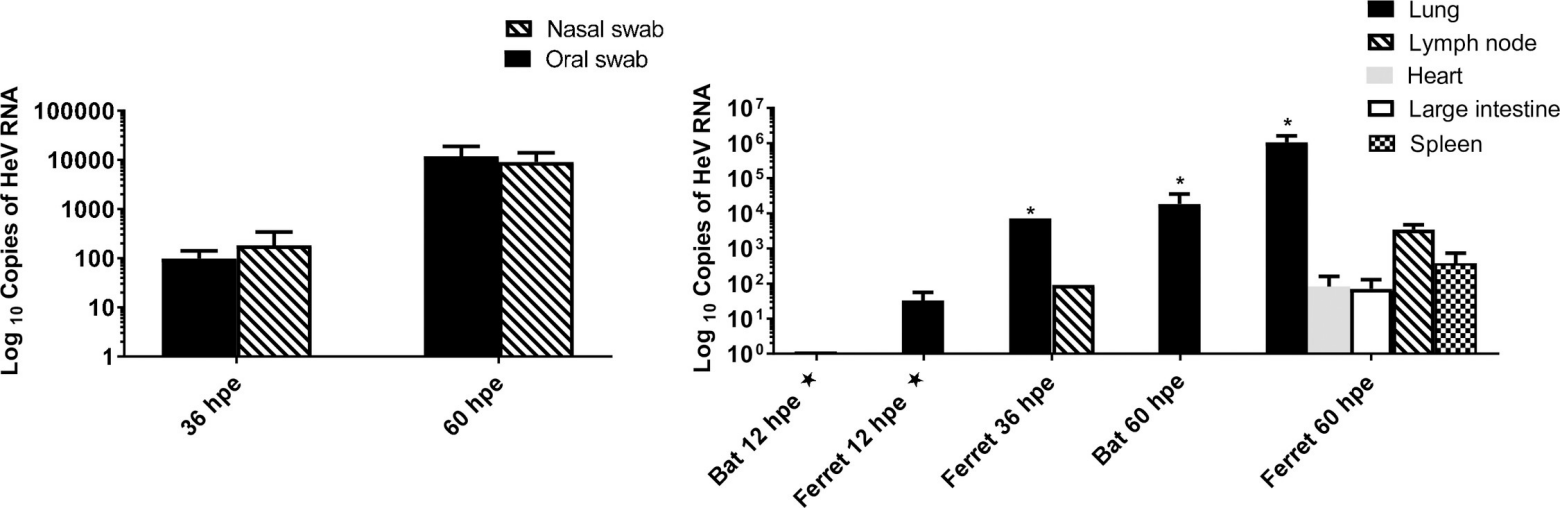
qRT-PCR detection of HeV M gene. (**A**) ferret swabs and (**B**) bat and ferret tissues (median + range). * designates tissues for which virus was re-isolated, ★ detections at 12 hpe may be derived from the inoculum.

Ferrets: Viral RNA was detected in the nasal and oral swabs of each ferret at 36 and 60 hpe (Fig 1A). All other oral, rectal and nasal swabs and urine samples were negative.

### Detection of HeV RNA and virus in tissues

**Fig 2:**
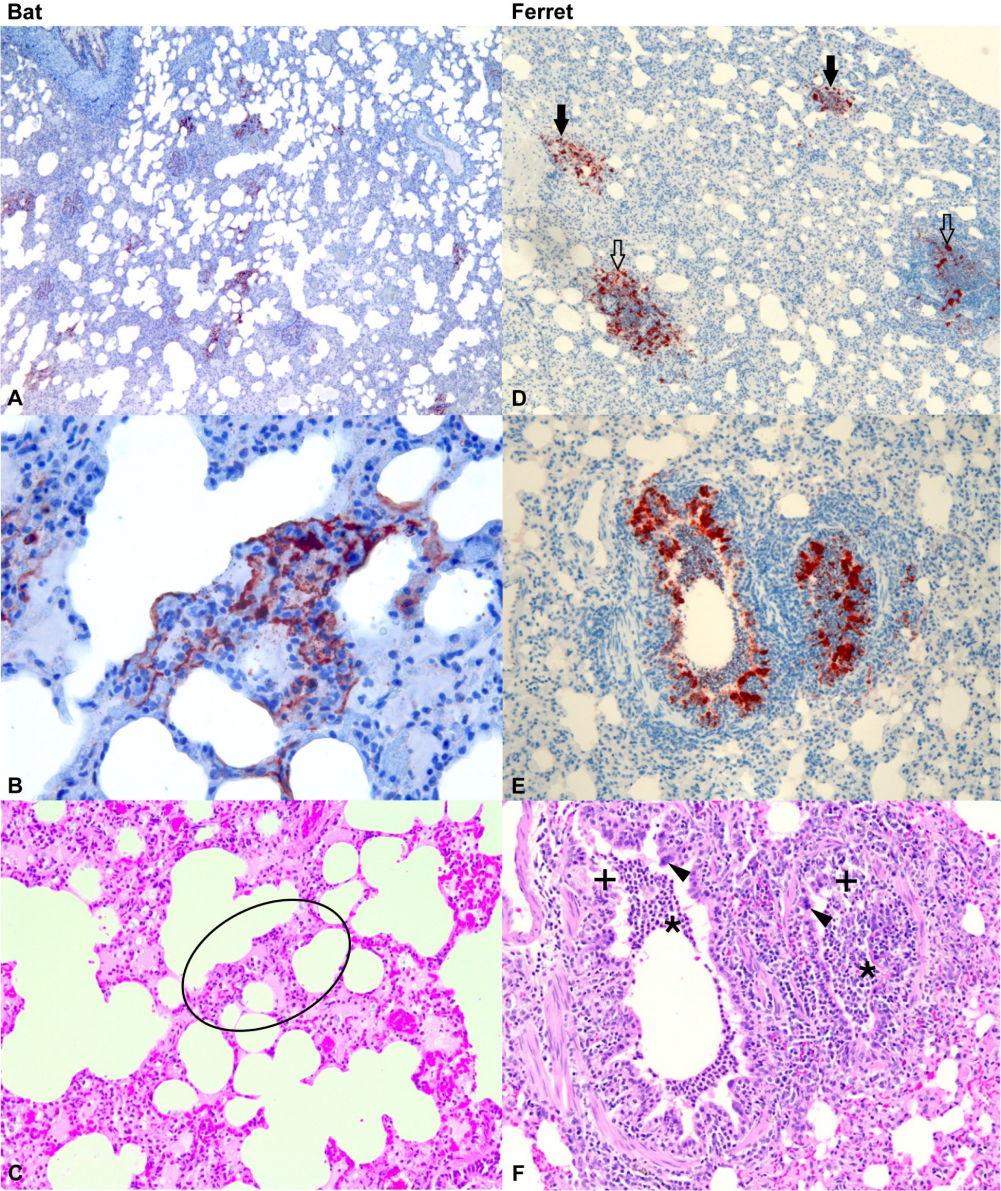
Histopathology of lung from infected bats and ferrets at 60 hpe. Immunohistochemistry for Hendra viral nucleoprotein (N) antigen (brick red colour) (A, B, D, E) and haematoxylin and eosin stain (C, F). (**A)** Bat lung showing multiple foci of viral antigen in interstitium. (**B**) Bat lung at higher magnification showing detail of antigen localization in alveolar wall. (**C**) Bat lung of the same area as B (within ring) showing the absence of abnormalities. (**D**) Ferret lung showing viral antigen in interstitial foci (filled arrows) and in association with bronchioles (open arrows). (**E**) Ferret lung showing viral antigen in bronchiolar epithelium and luminal cell material. (**F**) Ferret lung of the same locality as E, showing epithelial cell degenerative changes (+) associated with antigen, neutrophil accumulation in the airway lumen (*) and syncytia of the epithelial cells (arrowheads). A, B, C: bat B8-60; D, E, F: ferret F8-60.

*Bats*: At 12 hpe, low copy numbers of HeV RNA were detected only in the lung of one bat (B3-12); this may have been derived from inoculum. No viral RNA was detected in any of the bat tissues collected at 36 hpe. At 60 hpe viral RNA was detected in the lung of both bats sampled (Fig 1B); all other tissues were negative. Virus was re-isolated from lung tissue at 60 hpe from one bat (Fig 2, S4 Table).

Ferrets: Low copy numbers of HeV RNA were detected at 12 hpe in the lung only of two of the three ferrets; this may have been derived from inoculum. At 36 hpe, HeV RNA was detected in the lung of one ferret and lymph node of the other. By 60 hpe, HeV RNA was found in the lung, spleen, lymph node, heart and large intestine of both ferrets (Fig 1B); all other samples were negative. HeV was re-isolated from the lung of both ferrets at 60 hpe and from the lung of one of the two ferrets at 36 hpe (Fig 2, S4 Table).

### Histopathology and immunohistology

Bats: Immunohistochemistry for viral nucleoprotein (N) antigen was performed on multiple tissues in each animal. Tissues examined in most animals included brain, spleen, liver, kidney, heart, skeletal muscle, lung, thymus, lymph nodes, trachea, oesophagus, mediastinum, great vessels, small and large intestines, stomach, pancreas, urinary bladder, gonads, structures of the head (including cribriform plate, olfactory bulbs of the brain, nasal turbinates) and salivary gland. There were no remarkable findings in any of the bat tissues at 0, 12 or 36 hpe. Viral antigen was detected in the lungs of the two bats euthanized at 60 hpe with multiple small foci of viral antigen present throughout the interstitium of the lung (Fig 2A, 2B and 2C). There was no apparent tissue response associated with the antigen foci in either of the bats euthanized at 60 hpe (Fig 2C). All other tissues were within normal limits.

Ferrets: A corresponding set of tissues to those examined in the bats were also examined by immunohistochemistry in each ferret. There were no remarkable findings in any of the tissues collected from ferrets at 0 and 12 hpe and one ferret at 36 hpe. Viral antigen was detected in the lungs of ferret F6-36, euthanized at 36 hpe and in the lungs and other tissues of ferrets F8-60 and F9-60, euthanized at 60 hpe. In the lung, viral antigen was present mainly in bronchiolar epithelium and in occasional foci in the interstitium. In bronchiolar epithelium, viral antigen was associated with epithelial necrosis and syncytia development, mild mononuclear cell submucosal and peribronchiolar infiltration, and accumulation of neutrophils within the lumen (Fig 2D, 2E and 2F). In ferrets F8-60 and F9-60, small amounts of viral antigen were detected in the sub-capsular region of the mediastinal lymph nodes; it was present in two small foci in the epithelium of the nasal turbinates of ferret F8-60 and in the epithelium of both salivary ducts of ferret F9-60 (S1 Fig).

### Summary comments on infectivity of HeV inoculum for bats and ferrets

Both animal species were confirmed to have become infected by the inoculum on the basis of detection of viral RNA in bat lung (60 hpe) and ferret lung, retropharyngeal lymph node, spleen, heart and intestine (at 36 and/or 60 hpe); HeV isolation from bat lung (60 hpe) and ferret lung (36 and 60 hpe); and HeV antigen in bat lung (60 hpe) and ferret lung, mediastinal lymph nodes, nasal turbinates and salivary ducts (36 and/or 60 hpe). There was a higher likelihood of recovering HeV RNA from organs other than lung in ferrets (p = 0.0012), reflecting systemic spread of virus in that species. For the purpose of analysis of *IFN* and *CXCL10* transcripts collected at timepoints 12 and 36 hpe, we assumed that each animal sampled at those times had also been infected.

### Transcription of IFN and CXCL10 following HeV infection in bats and ferrets

qRT-PCR was performed to examine the transcription of type I (*IFNA*, *IFN beta [IFNB]*), type III (IFN lambda [*IFNL]*) IFNs and the chemokine, *CXCL10*, in lung and spleen tissues from bats and ferrets at 12, 36 and 60 hpe. Lung was chosen due to recovery of HeV RNA, antigen and virus from this tissue in both bats and ferrets at 60 hpe. Spleen was included as a representative immune tissue. *IFN*s were examined due to their role in the early innate immune response. *CXCL10* was included due to its role in the development of innate and adaptive immunity in concert with IFNs and evidence for its role in the pathogenesis of NiV infection other species [22,23].

### Type I IFN

**Fig 3:**
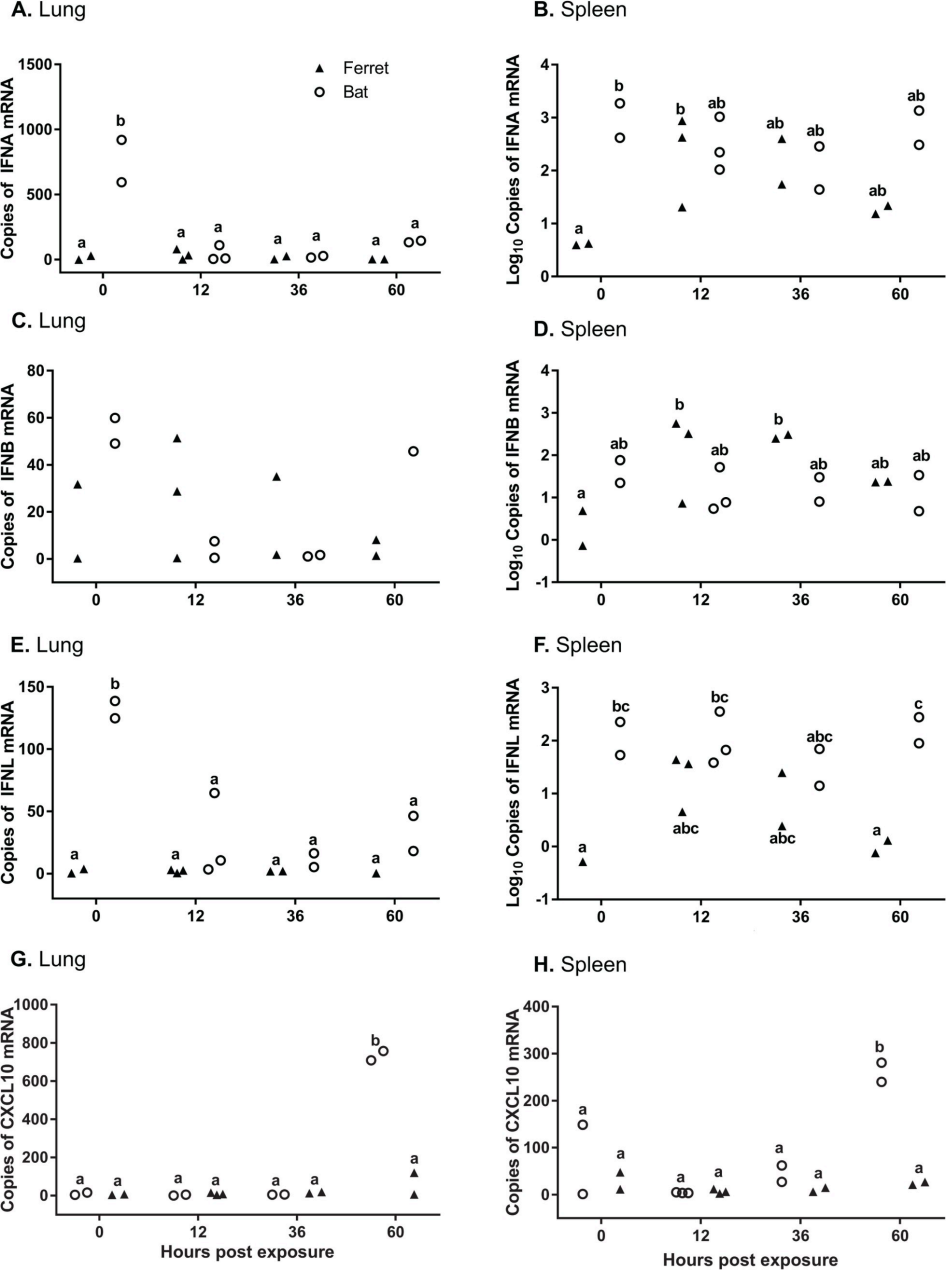
qRT-PCR of IFNs and CXCL10 in lung and spleen. Detection of *IFNA* in lung (**A**) and spleen (**B**), *IFNB* in lung (**C**) and spleen (**D**), *IFNL* in lung (**E**) and spleen (**F**), *CXCL10* in lung (**G**) and spleen (**H**) in bats and ferrets infected with Hendra virus. Real time PCR was used to assess gene expression in lung tissue at 0, 12, 36 and 60 hpe. mRNA copy number in lung and spleen tissue normalized to GAPDH. Different letters indicate mRNA expression that differs significantly (p<0.05) between samples.

Significantly higher levels of *IFNA* mRNA were observed in uninfected bat lung compared to infected bats and ferrets at 12, 36 and 60 hpe (p < 0.0001 to p = 0.003), and compared to uninfected ferrets (p < 0.0001) (Fig 3A). In the spleen, lower mean levels of *IFNA* were observed in uninfected ferret spleen compared to 12 hrs (p = 0.0350), and compared to uninfected bat spleen (p = 0.0077) (Fig 3B).

There was no significant effect of sample time or species on overall mean *IFNB* expression within the lung (Fig 3C). Higher levels of *IFNB* were observed in ferret spleen at 12 hpe (p = 0.0408) and 36 hpe (p = 0.0221) compared to uninfected ferret spleen (Fig 3D).

### Type III IFN

Higher mean levels of *IFNL* mRNA were observed in uninfected bat lung compared to bat and ferret lung at 12, 36 and 60 hpe (p = 0.0004 to p = 0.0015) and compared to uninfected ferret lung (p = 0.0002) (Fig 3E). There were higher levels of *IFNL* in the spleen of uninfected bats (p = 0.0155) and at 60 hpe (p = 0063) compared to ferrets. *IFNL* was significantly higher in bat spleen at 12 hpe compared to ferret spleen at 0 or 60 hpe (p < 0.05) (Fig 3F).

### CXCL10

There were higher mean levels of *CXCL10* observed at 60 hpe in bat lung compared to ferrets at 60 hpe (p < 0.0001), and compared to bat and ferret lung at all other times (p<0.0001) (Fig 3G). In the spleen, there were higher mean levels of *CXCL10* observed at 60 hpe in bats compared to ferrets at 60 hpe (p = 0.0003), and compared to bat and ferret spleen at all other times (p = 0.0011 to p < 0.0001) (Fig 3H).

### Proteomics analysis of HeV infected bat and ferret tissues

Due to the detection of viral RNA and antigen in bat and ferret lung at 60 hpe, and the observation of an inflammatory response in the lung of ferrets but not bats at this timepoint, LC-MS/MS analysis was performed on lung tissues collected at 60 hpe for detection of viral and host proteins. Proteomics was also performed on uninfected tissues to compare the effect on host protein regulation upon exposure to HeV.

### Detection of HeV proteins by mass spectrometry

**Fig 4:**
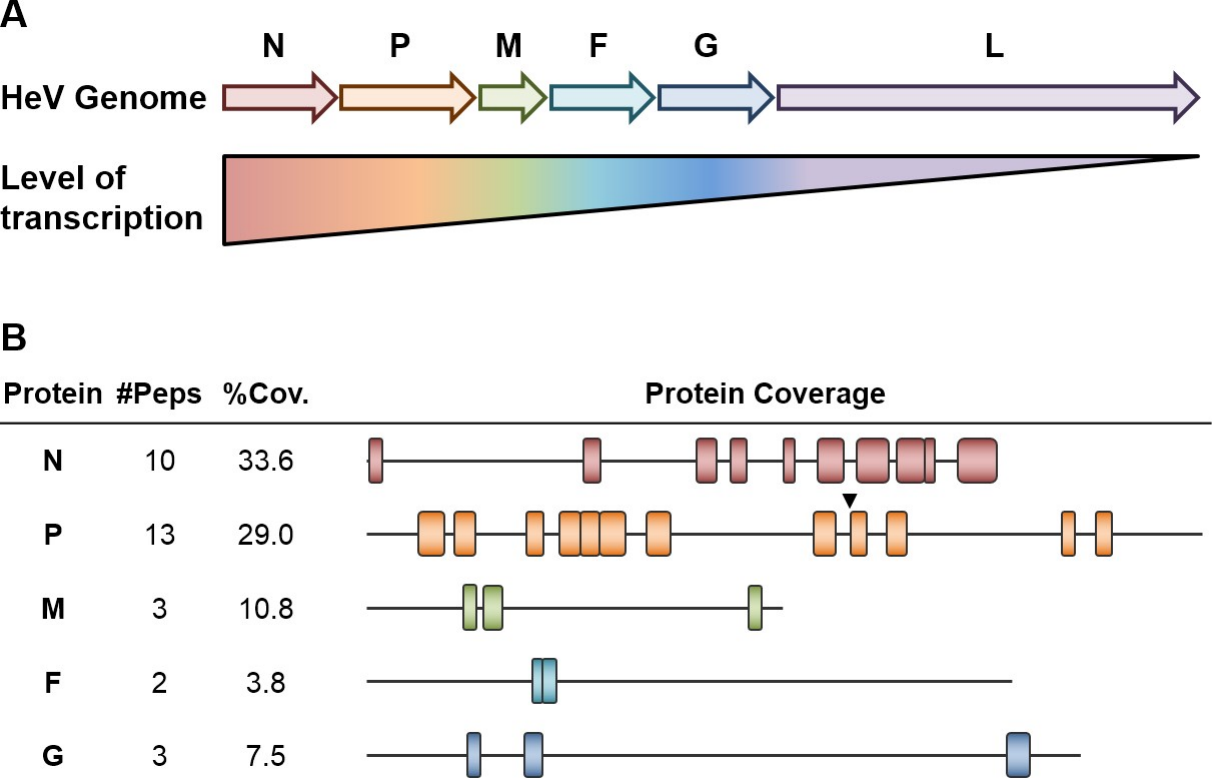
Detection of HeV protein products in infected ferret lung tissues using LC-MS/MS. (**A**) The HeV genome consists of six genes (3’-N, P, M, F, G, L-5’). A polar transcription gradient is formed where 3’ genes are transcribed more frequently than genes further downstream. These genes give rise to the six structural HeV proteins N, P, M, F, G and L. RNA editing of the P gene can also yield V and W protein and transcription of an alternative reading frame of the P gene produces C protein. (**B**) Using LC-MS/MS, five HeV structural proteins were detected. The number of peptides (#Peps) and percentage of protein coverage (%Cov) are listed. For each observed HeV protein, a schematic diagram representing the peptide regions of protein detected by LC-MS/MS is shown. The position and size of each peptide are to scale. The arrow represents the point at which the amino acid sequence of the P, V and W proteins differ downstream (position 405).

LC-MS/MS analysis identified HeV structural proteins, N, P, M, F, G, in both biological replicates of HeV-infected ferret lung tissues at 60 hpe but not in the bat lung tissues at this timepoint. A higher number of non-redundant N and P tryptic peptides were detected compared to M, F and G which is consistent with higher transcription levels of the 3’ located genes encoding N and P proteins [24] (Fig 4A). This number of HeV peptides also roughly coincided with the percentage of protein coverage (Fig 4B) which was calculated based on the identity and position of peptides detected by LC-MS/MS. The L protein, which is the least abundant HeV structural protein, was not detected by LC-MS/MS. It must be noted that although protein coverage correlated with the polar transcription gradient observed in HeV, this does not necessarily constitute protein abundance. Some proteins may have more accessible sites for tryptic digestion and some tryptic peptides may ionize more efficiently, thereby increasing the detected protein coverage. Quantitative proteomic techniques would have to be used to ascertain the absolute levels of HeV protein. It is believed that the increase in transcripts in the 3’ direction translates to an increase in protein abundance but no study has explicitly shown this yet.

Over 30 non-redundant HeV peptides were identified across both infected ferret lung replicates (S1 Table). Out of all HeV proteins detected, the P protein was detected with the highest number of non-redundant peptides (Fig 4B). The insertion of one or two guanines at an RNA editing site in the P mRNA transcript produces V and W proteins respectively [25]. The P, V and W proteins share the first 405 amino acids but downstream from this, the amino acid sequence differed for all three proteins. Of the 13 peptides originating from the P protein, the majority of these peptides were upstream of position 406. These nine peptides were not proteotypic meaning they could have originated from the P, V or W proteins.

Nevertheless, it is likely that these nine peptides were derived from the P protein as this protein is expressed at high levels and four P protein-specific peptides were detected downstream of position 405. Additionally, the V and W proteins are temporally regulated and are known to be present at a low abundance [25]. Furthermore, no proteotypic V and W peptides were detected. However, without further validation, the protein source of the nine peptides upstream of position 406 cannot be distinguished. Thus, it is possible that seven out of nine HeV proteins were detected in infected ferret lung samples.

### Detection of host proteins by mass spectrometry

To detect changes in host proteins, LC-MS/MS analysis was performed on uninfected (0 hpe) and infected (60 hpe) bat and ferret lung. Fold change for bats are indicated as BB1 (for B1-0/B8-60) and BB2 (for B2-0/B9-60) and for ferrets as FF1 (for F1-0/F8-60) and FF2 (for F2-0/F9-60) throughout the paper. A total of 3723 and 4355 differentially expressed proteins were identified in the bat datasets, BB1 and BB2 respectively and 3393 and 4542 differentially expressed proteins were identified in FF1 and FF2 respectively (S2 Table). There were 5487 non-redundant bat proteins and 6098 non-redundant ferret proteins identified by LC-MS/MS in the lung samples. This represents approximately 40% of both the bat and ferret proteomes and is, to our knowledge, the largest proteomics dataset available for bats and on par with previous proteomics datasets for ferrets [20]. Using a conservative cut-off of 3-fold, 820 unique bat proteins and 363 unique ferret proteins were assessed as upregulated and 806 unique bat and 197 unique ferret proteins were assessed as downregulated following infection. This cut-off was chosen based on the fold change expected to be above the normal range within an index population and based on previously published literature [26].

**Fig 5:**
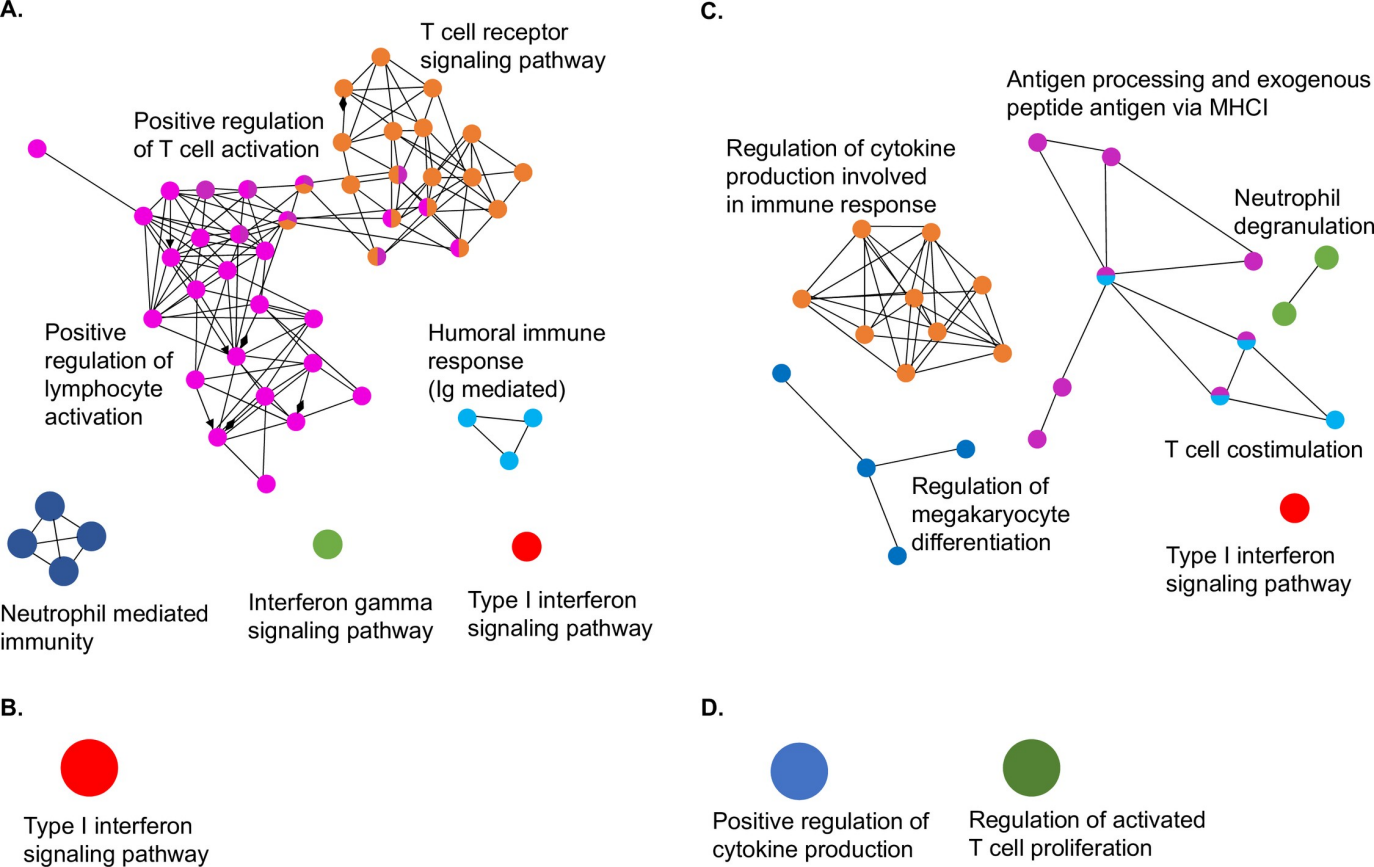
GO analysis of proteomics data showing upregulated pathways in bat and ferret lung. Upregulated pathways following HeV infection (60 hpe) compared to uninfected (0 hpe). Bat lung: (**A**) BB1 and (**B**) BB2. Ferret lung: (**C**) FF1 and (**D**) FF2.

Gene ontology (GO) analysis was performed on proteins detected in bat and ferret lung samples that were upregulated by at least 3-fold to enrich for immune pathways. A total of 766 and 62 proteins for bat BB1 and bat BB2 respectively and 283 and 88 proteins for ferret FF1 and ferret FF2 respectively were upregulated 3-fold or more and were used in this analysis. As shown in Fig 5A–5D, considerable variation was observed between and within each species. Bat BB1 and ferret FF1 displayed the highest number of enriched pathways following GO analysis of upregulated proteins.

Based on the GO analysis of immune pathways, subsets of proteins that were enriched in at least one dataset were compared between bats and ferrets. Table 1 shows the pathways that were analysed based on GO enrichment analysis. The list of proteins analysed in each pathway is shown in S3 Table. Of 12 pathways assessed, six were significantly more likely to be upregulated in bats compared to ferrets (neutrophil mediated immunity [p < 0.0001], IFNγ mediated signalling [p = 0.0033], positive regulation of T cell activation [p = 0.0001], T cell receptor signalling pathway [p<0.0001], humoral immune response mediated by circulating Ig [p = 0.0024] and positive regulation of lymphocyte activation [p < 0.0001]). A further two pathways were significantly more likely to be upregulated in ferrets compared to bats (neutrophil degranulation [p < 0.0001]; antigen processing and exogenous peptide antigen via MHC class I [p = 0.0008]) (Table 1). The remaining pathways were not significantly more likely to be upregulated in either species, including the type I IFN signalling pathway, which was enriched in the two bats and one of the two ferrets. T-cell co-stimulation, regulation of megakaryocyte differentiation and regulation of cytokine production were also not significantly more likely to be upregulated in either species.

**Table 1:** Pathway enrichment in bat and ferret lung proteomics identified by GO analysis.

Upregulated pathways	Downregulated pathways
*Neutrophil mediated immunity (GO:0002446)	*Activation of immune response involved in proteasome (GO:0002253)
*IFNγ mediated signaling pathway (GO:0060333)	*Myeloid activation involved in immune response (GO:0002275)
*Positive regulation of T cell activation (GO:0050870)	*Myeloid cell differentiation (GO:0030099)
*T cell receptor signaling pathway	^§^Activation of immune response involved in complement pathways (GO:0002253)
*Humoral immune response mediated by circulating Ig	^§^Complement activation (GO:0006956)
*Positive regulation of lymphocyte activation (GO:0051251)	Positive regulation of production of molecular mediator of immune response (GO:0002702)
^§^Neutrophil degranulation (GO:0043312)	Mast cell activation involved in immune response (GO:0002279)
^§^Antigen processing and exogenous peptide antigen via MHC class I (GO:0002479)	Positive regulation of myeloid cell differentiation (GO:0045639)
Regulation of cytokine production (GO:0002720)	Complement activation, alternate pathway (GO:0006957)
Type I IFN signaling (GO:0060337)	Complement activation, lectin pathway (GO:0001867)
Regulation of megakaryocyte differentiation (GO:0045652)	Negative regulation of leukocyte differentiation (GO:1902106)
T cell co-stimulation (GO:0031295)	

* Significantly different in bat compared to ferret

^§^ Significantly different in ferret compared to bat

**Fig 6:**
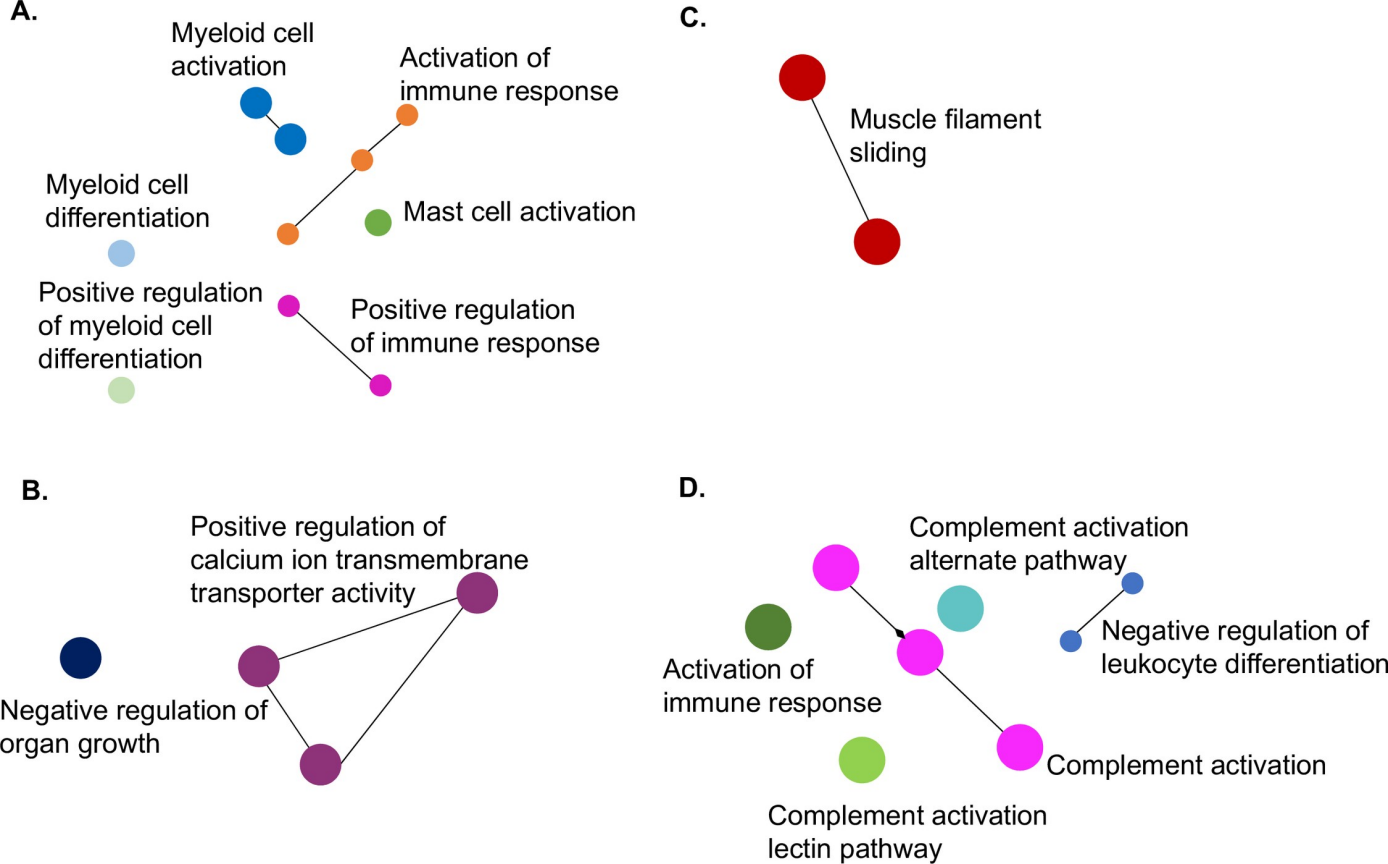
GO analysis of proteomics data showing downregulated pathways in bat and ferret lung. Downregulated pathways following HeV infection (60 hpe) compared to uninfected (0 hpe). Bat lung: (**A**) BB1 and (**B**) BB2. Ferret lung: (**C**) FF1 and (**D**) FF2.

A corresponding analysis was performed on the downregulated proteins detected by proteomics in bat and ferret tissues (Fig 6A–6D). A total of 732 and 82 proteins for bats BB1 and BB2 respectively and 67 and 131 proteins for ferrets FF1 and FF2 respectively were downregulated 3-fold or more and were used in this analysis. Bat BB1 and ferret FF2 displayed the highest number of enriched pathways following GO analysis of downregulated proteins. GO analysis resulted in the selection of eleven downregulated pathways for comparison between bats and ferrets (Table 1). A total of three were significantly more likely to be downregulated in bats compared to ferrets (activation of immune response involved in proteasome activation [p < 0.0001], myeloid activation involved in immune response [p < 0.001] and myeloid cell differentiation [p = 0.0002]). A further two pathways were significantly more likely to be downregulated in ferrets compared to bats (complement activation [p < 0.0001], activation of immune response involved in complement pathways [p = 0.0002]). The remaining six pathways were not significantly more likely to be downregulated in either bats or ferrets (Table 1).

## Discussion

In this study, acute infection with HeV was examined in Australian black flying foxes and ferrets to provide insights into the kinetics of early infection and innate immune activation. Unlike previous experimental infections that have used wild caught bats, the individuals used in the present study were born in captivity in a closed colony and were of known age and history at the time of experimental challenge, thus reducing the impact of capture and transfer into captivity or prior HeV infection on the immune response. Ferrets and bats were inoculated via the oronasal route using an isolate of HeV derived from an infected horse. Virus replication was detected in the lungs of both bats and ferrets. Differences in mRNA expression of *IFN*s and *CXCL10* revealed species-specific expression patterns across the time course of infection. Furthermore, global proteomics analysis of infected bat and ferret tissues provides insight into differences in immune pathway activation between the two species.

Viral antigen was undetectable in bat tissues at 36 hpe but was present in the lung interstitium at 60 hpe, with small foci of HeV N antigen identified by immunohistochemistry, although no associated tissue or inflammatory reaction was observed. Virus isolation confirmed replication of virus in this tissue. This is the earliest timepoint at which viral replication has been detected in bats following experimental infection. Previous studies have reported positive immunostaining for HeV in the blood vessels of the kidney, spleen, meninges and placenta with virus isolation from heart, buffy coat, kidney and spleen of *P*. *poliocephalus* bats at 10 days following subcutaneous exposure to HeV [13]. Oronasal inoculation of bats with HeV has been performed using *P*. *alecto* and *P*. *poliocephalus* bats, and in each case HeV antigen was not detected in any tissues collected at the study endpoint of 21 days post exposure (dpe) [12,27]. Prior to our study, no reports have looked for evidence of HeV replication earlier than 21 dpe in bats exposed by the oronasal route, despite this being the most likely route of natural infection. Our results provide evidence that HeV may arrive into the lungs of bats and result in a self-limiting infection. This pattern of viral replication is reminiscent of NiV infection in mice which develop a subclinical self-limiting lower respiratory tract infection following intranasal exposure to NiV [28].

Analysis of ferrets provided the opportunity to compare the replication of the virus in a susceptible model species. Previous studies have demonstrated that HeV infection of ferrets is associated with the establishment of a fever by 6 dpe and a humane endpoint is reached by 6 to 9 dpe [16]. Although the timepoints used in the present study were too early for the appearance of clinical signs of disease, we were able to determine some differences in the early pathology between the two species. These animals appeared to be in the early stages of infection, judging by the amount and distribution of antigen. Although similar amounts of antigen were seen in infected bat and ferret lung sections, in bats viral antigen was confined to lung interstitium, whereas in ferrets viral antigen was also identified in bronchiolar epithelium consistent with replication in those cells. There appeared to be minimal tissue and inflammatory response in bat lung, whereas in ferret there was a prominent inflammatory response and tissue degenerative changes.

Previous studies of HeV infection of ferrets challenged by oronasal inoculation have reported the lowest relative Ct level and highest proportion of virus isolations in the kidney, lung and spleen in ferrets euthanized at 6–9 dpe [16]. In the present study, viral RNA was identified in the lung, heart, large intestine, lymph node and spleen by 60 hpe. The absence of viral replication in the kidney of ferrets in our study suggests that this may be one of the last sites in which virus replication is established during a systemic infection. Similarly, Leon et al., [14] did not find HeV RNA in the kidneys of young ferrets (5–6 weeks old) at 1 dpe, although it was detected at 3 and 5 dpe.

To investigate the nature of the innate immune response, we compared the transcription of type I and III *IFN*s and the chemokine *CXCL10* in bat and ferret tissues. Although a number of studies have examined the host response of cell lines to henipavirus infections, this is the first report to describe the profile of *IFN*s in infected pteropid bat tissues and the earliest timepoint reported for ferrets [14,29,30,31]. Consistent with our previous observations, *IFNA* and *IFNL* were significantly higher in uninfected bats compared to uninfected ferrets, supporting evidence for constitutive activation of the pteropid bat IFN response [21]. The higher IFN expression may allow pteropid bats to respond more rapidly to infection. However, both type I and III *IFN*s were antagonized following HeV infection in bat tissues. Overall, these results are consistent with earlier observations of antagonism of the IFN response in bat cells *in vitro* [30].

The type I and III *IFN* response was also antagonized in the lungs of ferrets, but in the spleen, *IFNA* and *IFNB* increased significantly following infection, possibly reflecting the higher proportion of immune cells in this tissue. Leon et al., [14] reported the activation of interferon stimulated genes in HeV infected ferret lung at 3 dpe that remained active by 5 dpe. Thus, activation of IFN in ferrets may contribute to the inflammatory changes associated with infection in this species, even at the early timepoints examined in the present study. Similarly, during influenza virus infection of ferrets, unregulated proinflammatory cytokines and reduced expression of anti-inflammatory cytokines in the lower respiratory tract have been linked with increased virus transmission and severe disease [32,33].

CXCL10 is a potent chemoattractant for activated Th1 lymphocytes and natural killer cells and is believed to play a role in the development of innate and adaptive immunity in concert with type I and II IFNs [22]. CXCL10 has been associated with both protective and pathological infections depending on the infection and host immune status [22]. Evidence for a role of CXCL10 in NiV infection has been reported, with induction of *CXCL10* in NiV infected human endothelial cells and in the brains of patients that succumbed to NiV disease. In hamsters, which are a model for human NiV disease, upregulation of *CXCL10* closely correlates with viral antigen expression, initially increasing followed by a decrease in expression the day preceding lethal outcome of the infection [23]. The mRNA expression of *CXCL10* increased significantly at 60 hpe in bat lung and spleen tissues. Although the striking increase in the expression of *CXCL10* in bats is at odds with the outcome of infection in human patients, it may hint at a protective role in bats.

To obtain a global overview of the host response to infection, proteomic analysis was performed on bat and ferret lung tissue at 0 and 60 hpe. Lung was chosen as this was the only tissue in which viral antigen was detected at 60 hpe in both species. To our knowledge, this is the largest proteomics dataset reported for bats and is on par with previous proteomics datasets from ferrets [20]. These datasets provide valuable insights into the expression of host and viral proteins in species for which there are few antibody reagents. Gene ontology analysis revealed pathways that were upregulated and downregulated in bats and ferrets. Although no IFNs or cytokines were detected in our proteomics datasets due to their transient nature, GO analysis revealed that IFN signaling was upregulated at 60 hpe in the two bats and in one of the two ferret datasets. Similarly, a report describing the transcriptome of HeV infected ferret lung recently revealed extensive activation of interferon stimulated genes 3 dpe that remained active by 5 dpe [14]. Although the upregulation of IFN signaling pathways in bats contrasts with the antagonism of IFN transcription in HeV infected bats, it is possible that the constitutive expression of IFN in uninfected bat tissues accounts for some level of upregulation in signaling pathways even after infection.

Proteins contributing to pathways involving neutrophil mediated immunity, T cell mediated immunity (IFNγ mediated signaling, T cell activation and signaling and lymphocyte activation) and antibody mediated immunity (immune response mediated by circulating Ig) were significantly more likely to be upregulated in bats compared to ferrets. The enrichment of pathways associated with T cell activation in bats is consistent with the upregulation of *CXCL10* mRNA and suggests an important role for cell mediated immunity in bats. Although few studies have examined the cell mediated immune response of bats, proteomics analysis of peptides presented by HeV infected bat cell lines have previously provided strong evidence that this arm of the immune response may be critical for controlling infection in bats and warrants further study [34].

In ferrets, neutrophil degranulation and antigen processing pathways were significantly more likely to be upregulated compared to bats. Neutrophil activation, which encompasses the broader activities of neutrophils including phagocytosis, release of granules, secretion of cytokines and recruitment of other immune cells was also a feature of the upregulated proteins identified in bats. Neutrophil activation and degranulation play an important role in host defense but have also been linked to the pathology of various inflammatory conditions, including viral infections [35]. NiV infection in pigs is associated with neutrophil infiltration and may contribute to pathology associated with disease [36]. Similarly, higher upregulation of neutrophil degranulation which results in the exocytosis of proteases and inflammatory mediators in ferrets may contribute to pathology observed in this species. GO analysis revealed downregulation of pathways associated with complement activation in ferrets compared to bats. In contrast, complement activation has been reported to be a feature of transcriptome data from HeV infected ferrets at 1 and 5 dpe and therefore requires further investigation at the mRNA and protein level [14]. Similarly, other pathways identified in this study warrant further confirmation in larger numbers of animals.

## Conclusion

This study contributes additional information on the kinetics of viral infection in pteropid bats and ferrets and provides important comparative data on the differences in activation of the host response to HeV in species that respond with innocuous (bats) versus fatal (ferrets) outcomes. Although the innate immune response has been hypothesized to be key to the control of viral infection in bats, this study provides new data suggesting an important role for the cell mediated immune response in bats. Although constitutive activation of IFNs by pteropid bats may allow for a more rapid response, downregulation of this response may be just as important for avoiding immunopathology. In contrast, the activation of IFNs in ferret spleen after infection may contribute in part to the immune overactivation that ultimately leads to clinical disease and mortality. Due to the limited group sizes used in the present study, further experiments using larger numbers of each species, and/or the use of more targeted analytical techniques should be carried out to confirm these findings. The observations from the present study extend previous observations and provide directions for future investigation.

## Material and methods

### Animals

Nine juvenile flying foxes (five females and four males, aged 18 months) and nine male ferrets aged 12–18 months were used in this study. Juvenile flying foxes were sourced as follows. Wild caught pregnant female black flying foxes (*P*. *alecto)* were transferred into captivity at AAHL in August 2011. All females gave birth in October to November 2011 and mothers and pups were monitored regularly for changes in serum antibody to HeV. The decline of maternal antibody to HeV in the captive born pups from 1 to 12 months post-partum has been described previously: antibody was undetectable in pups at the time of experimental infection [37]. Ferrets were sourced from a commercial supplier (Whittlesea, VIC) and housed at the Werribee animal facility for 6 months prior to transfer to AAHL. Animals were acclimatized to the Biosafety Level 4 (BSL4) facility for five days prior to experimental infection.

### Ethics statement

Bats were caught and held under Queensland Environmental Protection Agency Scientific permit #WISP06386409 and Victorian Dept. of Primary Industries (DPI) Scientific permit #13909659; bats were imported to Victoria from Queensland under Victorian DPI Import permit #13894504. All animal experiments were approved by the CSIRO Australian Animal Health Laboratory (AAHL) Animal Ethics Committee (protocols 1474 and 1558) and were performed in strict adherence to guidelines dictated by the Australian Code of Practice for the Care and Use of Animals for Scientific Purposes.

### Animal accommodation, handling and biosafety

For the duration of the study, bats and ferrets were housed in a single room at BSL4. Room temperature was maintained at 22°C with 15 air changes per hour, humidity between 40–60%. Staff wore fully encapsulating suits with breathing apparatus while in the animal room. Bats and ferrets were introduced into the BSL4 room at the same time and housed in separate cages. Bats were housed individually in squeeze bottom cages (750 mm wide by 570 mm deep and 600 mm high). They were fed a variety of fresh fruit and provided with water *ad lib*. Ferrets were housed in pairs in cages that incorporated two “squeeze” compartments. They were fed a complete premium dry food and provided with water *ad lib*.

Before any manipulation, animals were immobilized with a mixture of ketamine HCl (Ketamil; Ilium, Smithfield, Australia; 5 mg/kg) and medetomidine (Domitors; Novartis, Pendle Hill, Australia; 50 mg/kg) administered by intramuscular injection. Where indicated, this was reversed by intramuscular antisedan at 50% of the medetomidine dose.

### Experimental design

Seven bats and seven ferrets were exposed to 30,000 TCID_50_ of the third passage in standard Vero cells of an equine isolate of HeV (Hendra virus/Australia/Horse/2008/Redlands [GenBank accession no. HM044317]). Inoculum was administered via mouth and nose drops (0.5ml per site). Droplets were delivered to the nose, allowing inspiration of droplets down the nostril, alternating sides with each droplet. Orally, droplets were distributed over the oral mucosa including the pharynx. Bats and ferrets were euthanized at 12 (n = 3), 36 (n = 2) and 60 (n = 2) hpe. The remaining two bats and two ferrets were used as unexposed controls and euthanized at 0 hpe. Clinical signs were assessed daily, and rectal temperatures and body weights were recorded at the time of exposure and the day of euthanasia. Animals were euthanized by the injection of pentobarbitone sodium (Lethabarb euthanasia injection, Virbac Australia) into the cardiac ventricles while under anaesthesia.

### Sample collection

Oral, nasal, and rectal swabs were collected prior to euthanasia and placed immediately into 2 mL viral transport medium (PBS containing 1% BSA with double strength antibiotic/antimycotic solution [Thermo Fisher Scientific]) and urine was collected by manual expression of the bladder; all specimens were stored at -80°C. At post mortem examination, blood, lymph node, salivary gland, lung, heart, liver, kidney, spleen, jejunum, ileum, large intestine, gonad and brain were collected for viral RNA detection, virus isolation, host gene detection, histology, immunohistology and proteomics. Tissue samples were collected into either 800 μL Magmax lysis/binding solution (Thermo Fisher Scientific) containing 1 mm stainless steel beads for RNA extraction or 10% neutral buffered formalin (Australian Biostain Pty. Ltd. Traralgon VIC) for 48 h prior to routine processing for histology. For proteomic analysis, tissues were homogenised in a ratio of 1:10 sample to SDT buffer (4% (w/v) SDS, 100 mM DTT, 100 mM Tris-HCl pH 7.6). Samples were then boiled at 95°C for 5 min and lysates were cleared by centrifugation at 13000 x *g* for 10 min.

### Detection of HeV RNA

Taqman RT-PCR assays targeting the HeV matrix (M) protein gene were used to detect viral RNA in blood, tissues, swabs and urine collected from bats and ferrets. Assays were performed using Superscript/Platinum TaqMan one-step qRT-PCR kit (Life Technologies) as previously described [38].

### Histology and immunohistochemistry

Tissues from euthanized bats and ferrets were collected into neutral-buffered formalin, processed and embedded into paraffin wax and sectioned to 5 μm. For histopathological interpretation, sections were stained with haematoxylin and eosin (HE); serial sections were stained in an immunohistochemistry test, according to methods described previously [39]. For the primary antibody, a rabbit antiserum directed against recombinant expressed NiV nucleoprotein described previously was used at a dilution of 1:1600 [40].

### Virus isolation

Virus isolation was performed as described previously. Briefly, supernatant dilutions from homogenized tissues (1/10 to 1/1000,000) positive for HeV genome were incubated on Vero cell monolayers and scored positive if syncytia, as a measure of viral cytopathic effect, were present after 6 days [16].

### Quantitative real time PCR (qRT-PCR) for detection of host genes

Quantitative RT-PCR was performed on lung and spleen tissues from bats and ferrets using primers targeting *IFNA*, *IFNB*, *IFNL*, *CXCL10* and *GAPDH* using the superscript RT-PCR kit (Life technologies). Primers for bat *IFNA*, *IFNB*, *IFNL2* and *GAPDH* have been described previously [21,41,42]. Primers for bat *CXCL10* (F: GAACTTCACGCTGTGTCTGC and R: TCTTTTTCATCGTGGCAATG) were designed based on sequence from the *P*. *alecto* genome (Genbank accession number ALWS01000000). Primers for ferret genes were designed based on sequences identified in the ferret (*M*. *p*. *furo*) genome (Genbank accession number AEYP00000000). Ferret primer sequences were as follows: *IFNA* (F: GAAGCAATACAGCCCTTGTG and R: CTGCTCCGCAATCTCTTATG), *IFNB* (F: TTTCTCCACCACGGTTCTTG and R: GTCCTTGAGGCAGTCTTTAG), *IFNL* (F: AGAAACCGGGACCTGAGACA and R: AGGTCAGCTCAGCCTCCAAG), *CXCL10* (F: TCCACGTGTTGAGATCATCG, R: CGCAGGATTCAGGCATCTTT) and *GAPDH* (F: ATGGTGAAGGTCGGAGTCAACGG and R: TTACTCCTTGGAGGCCATGTAGACC). Briefly, total RNA was extracted using the RNeasy minikit (Qiagen) and 5 μg of total RNA was reverse transcribed using Superscript III (Life Technologies) primed with oligo-dT as per the manufacturer’s instructions. Duplicate SYBR green real time PCR reactions were performed in a 25 μL reaction volume, containing 1 x EXPRESS SYBR green master mix (Life Technologies), 200 nM forward and reverse primers, and 20 ng template. Cycling parameters were 95°C for 10 min followed by 40 cycles of 95°C for 30 sec, 55°C for 30 sec and 72°C for 1 min, followed by melt curve analysis. Copy number was normalized relative to *GAPDH*.

### Filter aided sample preparation (FASP) tryptic digest of tissue proteins

As viral antigen was detected in lung tissue at 60 hpe in both bats and ferrets, this tissue was chosen to perform proteomics analysis for the detection of host and viral proteins. Lysate (400 μg of protein) from lung tissue of uninfected (0 hpe) and infected (60 hpe) animals was added to a Nanosep 10 kDa molecular weight cut-off filter (OD010C34; Pall) and processed using a filter-aided sample preparation (FASP) Protein Digestion Kit (Expedeon Inc) as described by manufacturers and by Wisniewski *et al*. [43]. All buffers were provided in the kit except for trypsin. Briefly, detergent was removed in a series of 8 M urea washes and cysteine residues were alkylated with the addition of iodoacetamide for 20 min in the dark. Proteins were washed twice with urea before being equilibrated with 50 mM ammonium bicarbonate. Proteomics grade porcine trypsin (T6567; Sigma) was added at a 1:80 (w/w) ratio of trypsin to protein and proteins were digested at 37°C overnight. The next day, tryptic fragments were eluted with the addition of 50 mM ammonium bicarbonate and 500 mM NaCl and particulates were removed by centrifugation at 16000 *x g* for 10 min. Each tissue sample was independently subjected to FASP tryptic digestion three times (experimental triplicates).

### Reversed phase-high performance liquid chromatography (RP-HPLC) separation of peptides

To increase the depth of detection, tryptic peptides were subjected to first-dimensional off-line RP-HPLC. Tryptic peptides were loaded onto a 50 mm x 4.6 mm internal diameter monolithic C18 RP-HPLC column (Chromolith Speed Rod; Merck) using an EttanLC HPLC system (GE Healthcare) with buffer A (0.1% (v/v) trifluoroacetic acid [TFA]) and buffer B (80% (v/v) acetonitrile [ACN], 0.1% (v/v) TFA) as mobile phases. Peptides were resolved over a gradient of 5% B to 40% B over 30 min. Fractions were vacuum concentrated at 40°C to 100 μL and every 7^th^ fraction was pooled (concatenate pooling).

### Identification of tryptic peptides by LC-MS/MS

Concatenated fractions were vacuum concentrated once more until approximately 5 μL and then reconstituted to a total volume of 30 μL with 0.1% (v/v) formic acid (FA), sonicated in a water bath for 15 min and centrifuged at 16000 x *g* for 10 min to remove particulates. Using an Ultimate 3000 RSLCnano HPLC (Thermo Scientific), each sample was loaded via a trap column (100 μm x 2 cm nanoViper PepMap 100; Thermo Scientific) in 2% (v/v) ACN, 0.1% (v/v) FA at a flow rate of 15 μL/min onto an analytical nanocolumn (75 μm x 50 cm PepMap 100 C18 3 μm 100Å; Thermo Scientific) at a flow rate of 300 μL/min. Peptides were separated using increasing concentrations of 80% (v/v) ACN, 0.1% (v/v) FA (buffer B) (2.5% B to 42.5% B over 20 min) and analysed with a Q Exactive Hybrid Quadrupole-Orbitrap mass spectrometer (Thermo Scientific). Up to 12 MS/MS spectra were acquired per cycle with maximum accumulation time of 50 ms and 100 ms for MS1 and MS2, respectively. To prevent multiple sequencing of the same peptide (dynamic exclusion), MS1 masses were excluded for 10 secs.

### MS data analysis

Samples were processed in two separate batches. Samples from bats B1-0 (0 hpe) and B8-60 (60 hpe) and ferrets F1-0 (0 hpe) and F8-60 (60 hpe) were first analyzed by LC-MS/MS. A second set of samples from bats B2-0 (0 hpe) and B9-60 (60 hpe) and ferrets F2-0 (0 hpe) and F9-60 (60 hpe) were analyzed separately. Due to differences in the LC instrumentation between the two runs, the raw data from each pair could not be cross-compared. Fold change between 0 and 60 hpe was therefore calculated within samples run at the same time for bats and ferrets.

To obtain peptide sequence information, mass spectrometry data files were converted using MSConvert (ProteoWizard 3.0) [44] and analysed using ProteinPilot software v5.0 (SCIEX). Data was searched against UniProtKB databases of either the *P*. *alecto* proteome or the *M*. *p*. *furo* proteome with the HeV proteome (HeV/Australia/Horse/2008/Redlands) appended. Proteins were considered to be present in a sample if two non-redundant peptides (above the 1% false discovery rate cut off) were detected by LC-MS/MS. Data analysis was automated by an R script.

Quantitation of host proteins was determined by label free quantitation (LFQ) using MaxQuant software v1.5.6.2 [45]. The LFQ value for each peptide was determined and statistical analysis was performed in the ancillary program, Perseus v1.4.1.3 [46]. Protein entries were retained only if LFQ values in all three experimental replicates at either time point (0 hpe or 60 hpe) were higher than zero. All retained LFQ values were log(2) transformed and a Student’s T test was performed to generate fold change values.

The mass spectrometry proteomics data have been deposited to the ProteomeXchange Consortium via the PRIDE [47] partner repository with the dataset identifier PXD017495.

### Gene ontology (GO) enrichment analysis

Bat and ferret UniProt accession IDs were converted into official gene symbols and then mapped to human GO slim terms using Generic GO Term Mapper [48]. GO enrichment analysis on differentially-expressed bat and ferret proteins was conducted using the ClueGO [49] and network maps were generated using Cytoscape [50]. A cut off of 3-fold (1.58 log fold change) up- or down-regulated was used for analysis of proteomics data. GO Term fusion, a process that groups parent-child terms based on similarly associated genes, was also applied to reduce redundancy in the number of GO biological processes shown. Many immune-related pathways were upregulated in the tissues interrogated but it was difficult to visualize them amongst the abundance of enriched metabolic and transport pathways. Thus, the upregulated target lists were reanalysed but this time only the “GO immune system processes” term was examined. This provided a more focused understanding of which immune-related pathways were enriched. Due to the small number of immune proteins represented in FF2, only the “GO biological process” was examined.

### Statistical analysis

All statistical analyses were performed using Graphpad Prism 8.10. *IFN* and *CXCL10* transcription data were analysed by ordinary two-way ANOVAs, with time and species as the independent variables. Raw data were used in these analyses, except for *IFN* within spleen where a log_10_ transformation was applied. For proteomic comparisons, the small numbers of replicates did not meet the criteria for valid Chi^2^ calculations on individual proteins. Accordingly, data from individual proteins belonging to the same recognised biological pathway (selected using ClueGo) were combined for each animal species, and the pathway data sets were subjected to contingency analysis using Fisher’s exact test. A similar approach was used to compare the likelihood of recovering HeV RNA from tissues other than lung between the two species.

## Supporting information

S1 Table Non-redundant HeV peptides identified by proteomics across in infected ferret lung replicates. (XLSX)

S2 Table Differentially expressed proteins identified in bats (BB1 and BB2) and ferret (FF1 and FF2) lung samples by LC-MS/MS. (XLSX)

S3 Table Protein lists generated from GO analysis of bat and ferret host proteins used in contingency analyses. (XLSX)

S4 Table Virus isolation from bat and ferret tissues. Only those tissues positive by qRT-PCR were tested by virus isolation.
(DOCX)

S1 Fig Immunohistochemistry for Hendra viral nucleoprotein antigen (brick red colour) in ferret tissues. (A) Mediastinal lymph node, showing viral antigen indicating infection of the lymphatic vessel walls (arrowheads) and invasion of viral antigen into the sub-capsular cortex of the lymph node. (B) Nasal turbinate showing focal viral antigen in nasal epithelial cells overlying sub-epithelial lymphoid aggregate. (C) Salivary duct, showing viral antigen in the duct epithelium and underlying tissue, associated with intense lymphohistiocytic inflammatory response in the duct wall (*). A, B: Ferret F8-60; C: Ferret F9-60.
(TIF)
