# Supplementary material for: Acute experimental infection of bats and ferrets with Hendra virus: Insights into the early host response of the reservoir host and susceptible model species
Source: PLoS Pathog. 2020 Mar 30;16(3):e1008412. doi: 10.1371/journal.ppat.1008412 (PMC7145190; doi:10.1371/journal.ppat.1008412)
Supplement: S4 Table — Only those tissues positive by qRT-PCR were tested by virus isolation. (DOCX) [file ppat.1008412.s004.docx]

**Table S4:** Virus isolation from bat and ferret tissues. Only those tissues positive by qRT-PCR were tested by virus isolation.

| **Sample** | **Isolation** |
| --- | --- |
| **Bats** | |
| B3-12 Lung | Negative |
| B8-60, Lung | Positive, 1:100 |
| B9-60, Lung | NA |
| **Ferrets** | |
| F3-12 Lung | Negative |
| F4-12, Lung | Negative |
| F5-12, Lung | Negative |
| F7-36 Lung | Positive, 1:100 |
| F7-36, Lymph node | Negative |
| F8-60, Lung | Positive, 1:100 |
| F9-60, Lung | Positive, 1:1000 |
| F8-60, Lymph node | Negative |
| F9-60, Lymph node | Negative |
| F8-60, Spleen | Negative |
| F9-60, Spleen | Negative |
| F8-60, Heart | Negative |
| F9-60, Heart | Negative |
| F8-60, Large intestine | Negative |
| F9-60, Large intestine | Negative |
