# Supplementary figures and images for: Acute experimental infection of bats and ferrets with Hendra virus: Insights into the early host response of the reservoir host and susceptible model species
Source: PLoS Pathog. 2020 Mar 30;16(3):e1008412. doi: 10.1371/journal.ppat.1008412 (PMC7145190; doi:10.1371/journal.ppat.1008412)

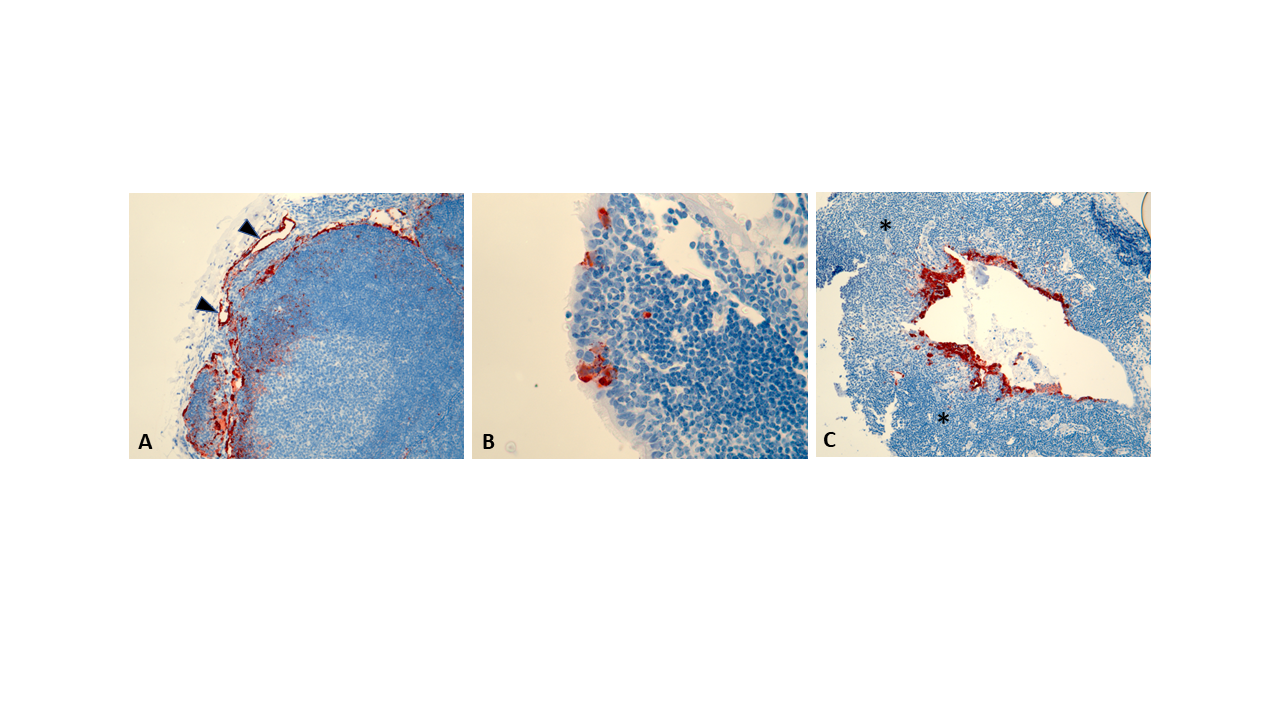

Supplement: S1 Fig — (A) Mediastinal lymph node, showing viral antigen indicating infection of the lymphatic vessel walls (arrowheads) and invasion of viral antigen into the sub-capsular cortex of the lymph node. (B) Nasal turbinate showing focal viral antigen in nasal epithelial cells overlying sub-epithelial lymphoid aggregate. (C) Salivary duct, showing viral antigen in the duct epithelium and underlying tissue, associated with intense lymphohistiocytic inflammatory response in the duct wall (*). A, B: Ferret F8-60; C: Ferret F9-60. (TIF) [file ppat.1008412.s005.tif]
